# Experimental-Duodeno Cholecystostomy

**Published:** 1885-11

**Authors:** J. McF. Gaston

**Affiliations:** Atlanta, Georgia


### EXPERIMENTAL DUODENO-CHOLECYSTOSTOMY.

BY J. McF. GASTON, M. D., ATLANTA, GEORGIA.

[CONTINUED FROM SEPTEMBER NUMBER.]

In compliance with a statement made at the close of my record of experiments upon fourteen dogs, in the August number of THE JOURNAL, I have to report that No. 7 of the series was subjected to a second laparotomy on August 1st, 1885. At my request, Dr. W. D. Bizzell performed this operation by making an incision in the median line, below the xyphoid cartilage, and, after verifying the attachment of the gall-bladder to the duodenum, attempted to ligate the common bile duct, which was attended with much difficulty on account of the adhesion of the liver to the pyloric orifice of the stomach, and depending chiefly on the sense of touch, he could not have full assurance of the result.

A third laparotomy was made in this case on October 3, 1885, by an incision extending obliquely across the epigastric region, below the line of union of the previous incisions, and I found the meso-colon adherent to the peritoneal lining so as to require its division throughout the entire extent of the opening. The liver was found adherent to the duodenum, and being separated by my fingers, I found the base of the gall-bladder firmly attached to the latter, and apparently without any collection of bile in the elongated sac, leading to the inference that it was kept drained through the artificial communication made by the loop of silk thread which had originally been passed through the adjacent walls of the gall-bladder and duodenum. As the common duct was now brought into view by the breaking up of the adhesions, at my suggestion Dr. Bizzell made another attempt at passing a catgut ligature around it, immediately below the entrance of the cystic duct.

The peritoneum was closed by continuous suture with Snowden's fine iron-dyed silk, No. 4, while the cutaneous incision was united by interrupted suture with No. 10 of the same thread.

October 5.—This animal appears to be recovering from the effects of the operation, and has taken food yesterday and to-day.

The subject of these experiments died Oct. 12, 1885, having survived the third laparotomy ten days. The autopsy revealed that the integument had not united, but the subcutaneous tissue and peritoneum closed up the abdominal cavity completely. The gall-bladder was moderately distended, but not tense, and the point of union with the duodenum was very well defined by intimate adhesion of their surfaces. Upon making an incision into the duodenum, there was observed opposite the cystic attachment, in the wall of the duodenum, an opening that admitted of the passage of a probe into the cavity of the gall-bladder, and through which bile escaped when pressure was made upon the sac. This communication was the result of a single loop of white silk thread passed through the walls of both on the 7th of July, 1885, and consequently has been preserved open something over three months, showing that such a fistulous communication is practicable. Further examination by Drs. W. D. Bizzell and K. C. Divine not only verified the existence of this artificial cholecysto-duodenal communication, but led to the inference that it had continued while the ductus choledochus was patulous, as a probe passed readily from the duodenal aperture of the common duct into one of the hepatic ducts, thus proving that the efforts to ligate it had not succeeded. It was evident in this case, as occurred also in

No. 9, that, on the last occasion, one of the hepatic ducts was mistaken for the common duct.

No. 9 of the series underwent a second laparotomy on August 15, 1885, by an opening in the linea alba between the xyphoid cartilage and the umbilicus. I found a firm attachment of the gall-bladder to the duodenum, but the distension of the sac made it very doubtful as to the fistulous communication of its cavity with that of the duodenum. The liver was adherent to the pylorus in such a manner as to intercept the view of the ducts, and I attempted by sense of touch to pass a catgut ligature around the common duct, but felt no confidence in the result.

There was not any evidence of extensive inflammatory action, beyond the local adhesions above noted, and the intestines presented no signs of previous peritonitis, though the entire mass had been upon the operating table for sometime during the first operation. Inner and outer suture of parietal incision done separately with the iron-dyed silk.

A third laparotomy was made in this case on September 25, 1885, embracing the line of the previous incisions, with a curved opening across the epigastric region, by Dr. Powell Walker.

After detaching the adhesions of the liver from the pylorus, I found an intimate union between the gall-bladder and duodenum, but upon making an incision of twc inches through the duodenal wall, immediately below the pyloric orifice of the stomach, only a cicatricial depression indicated the point where the silk thread had cut its way out. It was also observed that pressure upon the gall-bladder caused the discharge of bile from the natural entrance of the common duct, indicating my failure in the previous effort at ligation. I re-opened the artificial communication by passing a trocar through the depression in the septum between the walls of the gall-bladder and duodenum, making another effort to tie the common duct, which was still out of sight from the adhesions. The duodenal and the peritoneal incisions were closed with continuous catgut suture, and the external wound with four hair-lip suture pins, having points of interrupted suture with iron-dyed silk in the intervening spaces.

The subject of this experiment was found dead on the morning of September 30th, and on removing the jacket, the external wound was gaping throughout, the pins and stitches having cut out of the skin. But the peritoneal closure was complete. The suture of the duodenum had given way at the lower part and extravasation of the food had occurred to a limited extent. The opening between the sac and duodenum continued to afford an outlet for the bile. I had mistaken the hepatic duct for the common bile duct in my last attempt at its ligation, and thus perhaps aggravated the otherwise groin surroundings of the case, which lead to a fatal result.

Case No. 12 having died on August 2, 1885, an autopsy revealed that no attachment of the gall-bladder and duodenum existed. Whether the stitch had become untied and passed into the intestine, or whether it had cut its way out of the wall of the sac, and after ulcerated through the constricted wall of the duodenum, nothing could be determined, as no traces of its site could be discovered. There were adhesions of the liver to the duodenum and of the small intestines to the line of the abdominal incision, which was not entirely closed at the external surface, and there were indications of peritonitis within the peritoneal cavity. There was bile in the sac, and indications of its having an outlet by the natural channel into the alimentary canal. The points through which the silk thread had passed in the wall of the gall-bladder were most probably occluded by an adhesion of the left lobe of the liver over a portion of its surface, this surgical proceeding of nature having been noted in one of my first series of cases after a knot had become unloosed in an elastic suture.

This dog was subjected to ill usage by another animal in the kennel on various occasions, which may have torn the surfaces of the sac and duodenum apart before the action of the suture had established adhesive inflammation between them.

Second laparotomy on case No. 13, August 15, 1885, by incision below the xyphoid cartilage in the linea alba a little to the left of the first opening.

The adhesions prevented a view of the union of the gall-bladder and duodenum, but I was satisfied by the touch that they were firmly attached together by the silk loop which had been passed through their walls. I attempted to ligate the common duct with catgut without having it in view, but had no assurance of success. Died August 18th, and Dr. Bizzell, in my absence, made the autopsy, realizing that the common duct was not tied, and that the silk thread which had attached the gall-bladder to the duodenum had effected their intimate union, but had failed to secure an opening between them. A very rare incident in the operation of a tightened loop of thread was presented in the cutting through of the wall of the gall-bladder and its healing up while the seton-like stitch was still lying loosely in the inner part of the wall of the duodenum. It is evident that there must have been two orifices along the tract of the thread, at an earlier period of the constriction of the enclosed tissues, which would have continued patulous had the common duct been occluded so as to force the bile from the sac into the duodenum. But as there was no *vis-atergo* these fistulous openings closed, and the tissues became consolidated completely.

Case No. 15, on October 12, 1885, a black and tan dog being etherized, I made an incision across the epigastric region, and with the assistance of Dr. W. P. Nicolson proceeded to explore the abdominal cavity. Having made an opening in the linea alba below the umbilicus in this animal on the 24th of July, with the co-operation of Dr. Henry Wile, I had excised twelve inches of the ileum, and attached the edges of the intestinal canal by thirteen silk stitches, and now, after two and a half months, the restoration of the bowel was found complete, with some adhesions to adjacent coils of intestine.

I proceeded to ligate the common bile duct with catgut, and passed a stitch of white silk thread through the walls of the gall-bladder and duodenum, so as to hold them in contact, while with the shoe-maker's punch a circular segment was removed from each, and the borders united by interrupted iron-dyed silk suture. This animal died in less than 24 hours, and the autopsy on the 14th showed the complete occlusion of the common duct by the ligature, but the stitches in the opening between the gall-bladder and duodenum had given way at one point so that bile and chyme escaped into the abdominal cavity, inducing some peritonitis.

There was evidence of extension of inflammatory action to the intestinal canal, and the contents of the ileum, when divided for removal of the re-united portion, were tinged with blood. The speedy fatal result was doubtless from shock.

An examination of the primary and secondary operations upon these fifteen dogs, in connection with my experiments upon the six dogs last year, is calculated to impress the reader with serious apprehension of the safety in undertaking this cholecysto-duodenal communication upon the human being. But there is an element of risk from the acute traumatic inflammation in the normal state of the parts in these several experiments, which does not enter into the calculation when an operation is called for by the pre-existing obstruction of the common bile duct in man. The slow progress of the impediment to the outlet of the bile by the gradual accretion of gall-stones, or the subacute inflammation of the walls of the ducts diminishing the flow by peacemeal, leads to changes which must give a great degree of tolerance for such an operation, and there is not that insult to the vital organization which ensues in the revolutionary action of a sudden interruption of the biliary discharge. We have to remedy what exists as a cause of long-standing trouble in man by an operation which must alleviate instead of aggravating the concomitant disorders; and the risk to life cannot be enhanced by making this opening between the gall-bladder and duodenum.

